# A practical approach for regulatory acceptance of virtual control groups: vehicle pooling and biological relevance assessment

**DOI:** 10.3389/ftox.2026.1888158

**Published:** 2026-09-04

**Authors:** Jiwon Kim, Yoongi Kim, Daeui Park, Sang Kyum Kim, Myung-Gyun Kang

**Affiliations:** 1 College of Pharmacy, Chungnam National University, Daejeon, Republic of Korea; 2 Center for National Toxicology AI and Data, Korea Institute of Toxicology, Daejeon, Republic of Korea; 3 Center for New Drug Development Support, Korea Institute of Toxicology, Daejeon, Republic of Korea

**Keywords:** biological relevance, historical control data, reduction, refinement, repeated-dose toxicity, replacement, vehicle pooling, virtual control group

## Abstract

The implementation of virtual control groups (VCG) to replace concurrent control groups (CCG) in toxicity studies is challenged by the need for strict covariate matching. This is particularly true in diverse administration vehicles, which severely fragment historical control data (HCD). To overcome this limitation, this study consolidated region-specific HCD from physicochemically similar aqueous vehicles (saline, distilled water, and 0.5% methylcellulose) into a site-specific pool for 13-week rat studies in the Republic of Korea, thus securing a sufficiently powered VCG pool. To rigorously evaluate VCG reproducibility beyond strict statistical thresholds (e.g., p < 0.05), a category-based assessment framework was introduced as a validation methodology focusing on the direction and magnitude (e.g., percentage change) of alterations. Although body and organ weights showed high statistical agreement between VCGs and CCGs, the exact reproduction of statistical significance for clinical pathology parameters was relatively low (25%–40%) owing to limited sample sizes and inherent biological noise. Nevertheless, the category-based assessment demonstrated 100% reproducibility based on the biological relevance of all evaluated clinical pathology parameters. Crucially, the VCG-based analysis confirmed that the study director’s fundamental toxicological interpretations and target organ signals remained robustly preserved. Overall, pooling HCD across similar vehicles resolves sample size constraints, and assessing VCG reproducibility via biological relevance provides a practical framework to enhance regulatory acceptance.

## 1 Introduction

The implementation of the replacement, reduction, and refinement (3Rs) principle has become a primary ethical and scientific objective in non-clinical safety assessments. The concept of a virtual control group (VCG) has recently gained substantial traction (Golden et al., 2023; Wright et al., 2023). A VCG utilizes historical control data (HCD) collected from standardized legacy *in vivo* studies to partially or completely replace concurrent control groups (CCG) in newly designed toxicity evaluations (Golden et al., 2023; Steger-Hartmann et al., 2025; 2020). The widespread adoption of standardized terminologies, such as the Standard for Exchange of Nonclinical Data (SEND), along with large-scale data-sharing initiatives, such as eTRANSAFE and VICT3R, has provided a foundational infrastructure to generate highly curated HCD pools for VCG implementation (Steger-Hartmann et al., 2025; Wright et al., 2023). The successful application of VCGs has the potential to reduce overall animal use by up to 25% in standard systemic toxicity studies, thereby mitigating ethical concerns and supply constraints (Steger-Hartmann et al., 2020; Wright et al., 2023). Despite their immense potential, routine implementation and regulatory acceptance of VCGs face two major practical and methodological hurdles. The primary challenge in generating a reliable VCG is the severe fragmentation of the available HCD pool caused by the use of diverse administration vehicles. Strict matching of fundamental covariates, such as species, strain, age, sex, and test facility, is intrinsically required to avoid baseline-driven biases (Wright et al., 2023). However, additionally enforcing exact vehicle matching drastically reduces the available sample size and hinders the construction of sufficiently powered VCGs (Li et al., 2024). Therefore, overcoming this vehicle-driven data limitation without compromising the physiological comparability is a critical prerequisite for the practical deployment of VCGs.

The second challenge is the traditional reliance on simple statistical significance (p-values) for evaluating VCG reproducibility. This approach has proven inadequate, particularly for clinical pathology endpoints (Gurjanov et al., 2024). Gurjanov et al. (2024) and Andaya et al. (2024) revealed that the exact reproduction of statistically significant findings between CCGs and VCGs is relatively low. This often leads to false positives or inconsistent interpretations. This discrepancy stems primarily from the inherent biological variability of animals, pre-analytical variables not captured in SEND, and statistical artifacts generated by evaluating numerous parameters without rigorous multiplicity adjustments (Gurjanov et al., 2024; Steger-Hartmann and Clark, 2023). Toxicological safety assessments fundamentally rely on a weight-of-evidence approach that integrates the magnitude of change, dose dependency, and correlative histopathology rather than isolated statistical thresholds. Therefore, evaluating VCGs solely based on p-values may distort the actual toxicological profile and hinder regulatory confidence (Adedeji and Naor, 2025; Wright et al., 2023).

To address these critical gaps and facilitate regulatory acceptance of VCGs in systemic toxicity studies, this study proposes a practical and biologically relevant evaluation framework. First, to secure a sufficiently powered VCG pool by overcoming data fragmentation, the study investigated the feasibility of pooling HCD by restricting the data to physicochemically similar aqueous vehicles (saline, distilled water [DW], and 0.5% methylcellulose [MC]). Subsequently, to validate the reliability of the generated VCGs in legacy 13-week repeated-dose toxicity studies, the evaluation was extended beyond the conventional statistical significance approach based on p-values. Although p-values are useful statistical metrics for supporting scientific decisions, relying solely on these values is often inadequate in toxicity studies. This is particularly true when sample sizes are small or specific endpoints exhibit high biological variability.

Therefore, this study introduced a novel category-based assessment to evaluate directional changes and percentage changes in variations. By applying this comprehensive approach, the study aimed to confirm the reproducibility of VCGs against conventional CCGs from a toxicologically meaningful perspective. Finally, the study aimed to verify whether VCG-based analysis could successfully reproduce the study director’s ultimate toxicological interpretations regarding test-article relatedness and target organ identification, which form the fundamental basis for toxicological evaluations. This will determine the robustness of VCGs as a practical alternative to concurrent controls.

## 2 Materials and methods

### 2.1 Data sources, selection, and characterization of the HCD pool

Individual animal data for the control groups were obtained from the HCD of the Korea Institute of Toxicology (KIT). All studies from which the HCD were derived were conducted in compliance with the Korean Animal Protection Act and the Laboratory Animal Act, approved by the Institutional Animal Care and Use Committee, and adhered to Good Laboratory Practice standards. The studies were conducted between 2017 and 2021 in accordance with the OECD Test Guidelines (e.g., TG 408), the ICH M3 (R2) guideline, or both. Legacy studies included in the HCD pool were conducted using Sprague-Dawley rats (Orient Bio Inc., Seongnam, South Korea) aged approximately 6–7 weeks at the initiation of dosing. All animals were housed in a controlled environment (temperature: 22 °C ± 3 °C, relative humidity: 50% ± 20%, and a 12-h light/dark cycle) with *ad libitum* access to a standard rodent diet. The test items and vehicles were administered via oral gavage with the dose volume for each animal calculated based on its most recently measured body weight. Data were collected in the SEND format. To integrate historical control data and the generation of VCGs, the original clinical pathology parameters were mapped to the SEND terminology. However, for clarity and readability, standard clinical pathology abbreviations have been used throughout the manuscript.

Ultimately, the constructed HCD pool comprised data from 428 animals (214 males and 214 females) derived from 14 independent legacy studies (Supplementary Table S1). The sample size per control group varied between 10 and 20 animals per sex.

**FIGURE 1:**
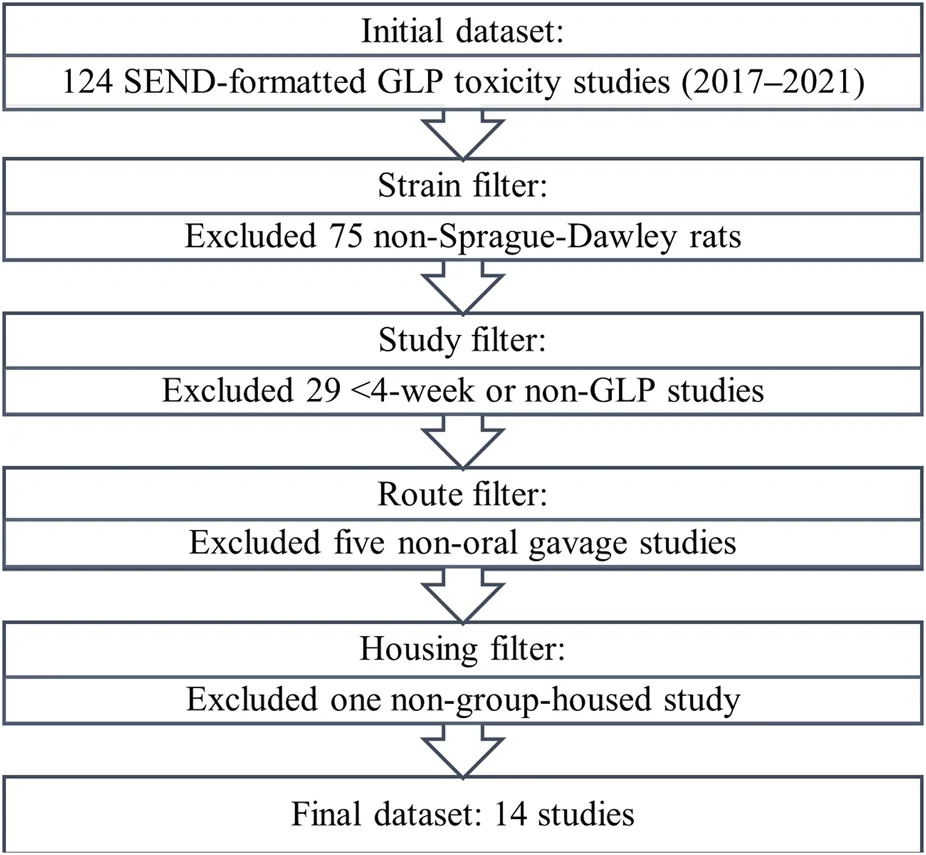
Historical control data (HCD) selection process. Abbreviations: SEND, Standard for Exchange of Nonclinical Data; GLP, Good Laboratory Practice.

Quantitative variables, including body weight, hematological parameters, clinical chemistry parameters, and absolute organ weights, were extracted. The systematic HCD selection process is shown in Figure 1.

A two-step data selection process was employed to establish the VCGs. First, an initial broad HCD pool comprising 4-, 13-, and 26-week studies was utilized to assess the impact of vehicle type on toxicological endpoints and to justify the pooling of aqueous vehicles (saline, DW, and 0.5% MC). Subsequently, to generate VCGs to replace CCGs in the 13-week legacy studies, a specific subset was derived from this pooled aqueous vehicle group. This subset was strictly limited to 13-week studies (or data measured at the 13-week timepoint) to ensure precise experimental matching.

### 2.2 Impact analysis of vehicle types

To evaluate the influence of vehicles on each toxicological endpoint, six types of vehicles were analyzed: saline, DW, 0.5% MC, 0.5% carboxymethyl cellulose (CMC), a specific vehicle provided by the study sponsor (hereinafter referred to as “Formulation”), and 1% Tween 80. Body weights, clinical pathology parameters, and absolute organ weights were compared between the groups. A Spearman’s rank correlation analysis using individual animal data was performed to determine the feasibility of pooling data across different vehicle types (detailed statistical procedure are described in Section 2.5).

### 2.3 Selection of legacy studies

Two legacy studies were selected from the previously established vehicle-matched HCD subset (comprising seven 13-week oral toxicity studies using highly correlated aqueous vehicles) to validate the reproducibility of the VCG-based analysis. These studies were selected because their original CCG-based interpretations demonstrated clear, test-item-related alterations in clinical pathology parameters. Selecting studies with such positive findings in quantitative clinical pathology provided a reliable reference to accurately evaluate whether the VCG approach could successfully reproduce these toxicological signals.

### 2.4 VCG generation via random sampling and pre-processing

To generate a distribution of virtual controls, animals were extracted from the pooled HCD dataset by random sampling without replacement. This process was repeated 100 times to minimize potential sampling bias from a single random draw, with the sample size of each VCG set adjusted to match the number of individuals in the corresponding treatment groups in the respective legacy studies (Gurjanov et al., 2024).

Preclinical toxicity studies operate under highly standardized Good Laboratory Practice (GLP) and international guidelines (e.g., OECD, ICH M3 (R2)). In our study, we strictly matched multiple fundamental covariates: the data originated from a single test facility, using the same species (rat), strain (Sprague-Dawley), and age (6–7 weeks old), sourced from the same supplier, under strictly controlled environmental conditions (temperature, humidity, diet), and utilizing studies initiated between 2017 and 2021. Importantly, our study took the extra step to tightly control the administration vehicle by restricting the pool exclusively to physicochemically similar aqueous vehicles.

To further align the baseline status between the VCGs and dose groups and to reflect the actual *in vivo* pre-dose randomization process, the dataset was additionally filtered using the initial body weight on Day 1, a practice well-supported in recent VCG literature (Andaya et al., 2024; Golden et al., 2023). Specifically, individuals were selected from the vehicle subset if their day 1 body weights fell within two standard deviations of the mean body weight of the corresponding treatment groups in the legacy study (Gurjanov et al., 2024).

### 2.5 Statistical analysis and software

Data curation and statistical analyses were performed using Python (version 3.12.2) and Microsoft Excel 2018, respectively.

- *Correlation analysis:* To evaluate the equivalence of the baseline across different vehicle groups, a Spearman’s rank correlation analysis was performed. Specifically, for each evaluated quantitative endpoint, individual animal data were utilized to calculate pairwise Spearman’s rank correlation coefficients (ρ) between the different vehicle types. This approach generated correlation matrices for each endpoint to assess the concordance of their baseline profiles. A correlation coefficient of ρ > 0.9 was interpreted as a strong correlation, providing the statistical justification for the consolidation of specific aqueous vehicles.
- *Significance testing:* To ensure direct comparison, the statistical methods used in the VCG-based analysis were identical to those employed in the original legacy studies. Specifically, a one-way analysis of variance or the Kruskal-Wallis test was performed, followed by Dunnett’s test or Dunn’s test, respectively.
- *Aggregation of statistical results via majority vote:* Because the VCG generation process involves 100 random iterations, evaluating a single parameter yields 100 distinct statistical results. To determine the final statistical significance for each parameter, a “majority vote” approach was employed, as previously described and validated by Gurjanov et al. (2024). Specifically, an overall result was considered statistically significant only if the exact same direction of significance (i.e., a consistent increase or decrease) was observed in at least 50 out of the 100 iterations. Furthermore, to report a single representative value for the newly generated VCG pool, the final descriptive statistics were derived from the medians of the 100 individual means and standard deviations.

### 2.6 Evaluation of reproducibility

The reproducibility of the results obtained using VCG were compared with those obtained using CCG and evaluated from three complementary perspectives.

- *Statistical significance agreement:* Individual parameters were classified into five groups based on the consistency of their p values between the VCG and CCG analyses. These groups were defined as follows: consistently significant, consistently non-significant, inconsistently significant, inconsistently non-significant, and inversely significant (Gurjanov et al., 2024).
- *Category-based assessment for biological relevance (direction and magnitude):* To overcome limitations of strict statistical thresholds (e.g., p ≤ 0.05) a category-based assessment was implemented. In nonclinical toxicity studies, determining the biological relevance of a finding relies fundamentally on comparing the magnitude of change against the natural biological variability of the animal population. Specifically, the relative magnitude of change compared with that of the respective control group was calculated and classified into four tiers (<25%, 25%–50%, 50%–100%, ≥100%). Based on direction, magnitude, and statistical significance, parameters were assigned to five reproducibility categories (Table 1). The parameter-specific physiological range (<2 CV%) was applied as the biological threshold for Category 4 (Supplementary Table S10). These CVs (calculated as standard deviation divided by the mean) were derived from internal historical data of 160 male and 160 female Sprague-Dawley rats from 13-week oral toxicity studies conducted during the data collection period. Because verifying whether a biological measurement falls within the Mean ±2 SD of normal individuals over a specific period is a standard toxicological procedure to define biological background noise, any deviation within this <2 CV% range was interpreted as normal physiological fluctuation rather than a reproducibility failure. Ultimately, the rate of reproducibility was defined as the percentage of parameters falling into categories 1–4, where the responses were considered sufficiently consistent and within acceptable biological limits.
- *Consistency with study director’s (SD) interpretation:* We acknowledge that in biological *in vivo* systems, completely eliminating inter-study variance is practically unattainable due to inherent biological noise and unmeasured preanalytical factors (e.g., minor lot-to-lot diet differences or handling stress), even under strict GLP standardization (Gurjanov et al., 2024; Steger-Hartmann et al., 2020). The fundamental objective of our study was to demonstrate that the VCG approach can successfully reproduce the test-article-related adverse effects and ultimate toxicological interpretations despite inevitable baseline variations. Rather than relying solely on simple statistical significance, which is highly sensitive to minor inter-study variations, we introduced the category-based assessment for biological relevance. Accordingly, the extent to which the VCG-based analysis reproduced the ‘meaningful changes’ described in the original study reports was assessed. This assessment focused on whether the VCG results aligned with the SD’s descriptions (e.g., ‘3-fold increase in ALT’) in terms of direction, magnitude, dose-dependency, and toxicological relevance.

**TABLE 1:** Definitions of reproducibility categories for biological relevance assessment.

Category	Description
1	Consistent direction and magnitude of change with identical statistical significance
2	Consistent direction and magnitude of change, but differing in statistical significance
3	Consistent direction of change, but differing in both magnitude and statistical significance
4	Opposite direction in some or all doses, but the percentage of change is within the parameter-specific coefficient of variation (CV) range of the internal historical control data (HCD)
5	Non-reproducible (inconsistent direction, magnitude, and significance)

## 3 Results

### 3.1 Feasibility of vehicle-based HCD integration to overcome covariate limitations

Fundamental covariates, such as strain, age, and test facility, must be strictly matched when generating VCGs from HCD. However, additionally enforcing exact vehicle matching severely limits the available data pool because of the diverse vehicles used across studies. To overcome this limitation, the integration of data from different vehicle types showing similar overall correlations across endpoints has been proposed. To this end, a Spearman’s rank correlation analysis was performed on toxicological endpoints from six vehicle types: saline, DW, 0.5% MC, 0.5% CMC, Formulation, and 1% Tween 80. The analysis demonstrated that the overall correlation coefficients ranged from 0.65 to 1.00 (median: 0.90, interquartile range [IQR]: 0.85–0.99) for males (Supplementary Figure S1) and from 0.66 to 1.00 (median: 0.93, IQR: 0.85–0.99) for females (Supplementary Figure S2). A detailed review of the correlation matrices revealed that saline, DW, and 0.5% MC consistently exhibited high inter-vehicle correlations (mostly ρ ≥ 0.94) across major toxicological endpoints, including sequential body weight measurements (e.g., days 8–29) and key clinical pathology markers, such as albumin (ALB), alanine aminotransferase (ALT), aspartate aminotransferase (AST), and activated partial thromboplastin time (APTT). Conversely, specific vehicles, such as Formulation and 1% Tween 80 showed relatively lower correlation coefficients (e.g., ρ = 0.74–0.77) than those of the aqueous vehicles in certain parameters, such as body weight.

The high inter-vehicle correlations indicated that the type of vehicle had a limited impact on the toxicological endpoints. Specifically, saline, DW, and 0.5% MC consistently showed stronger correlations than those of Formulation and 1% Tween 80. In addition, they share similar physicochemical properties with aqueous-based vehicles. The aqueous-based vehicles exhibit similar physicochemical properties. For these reasons, studies using saline, DW, or 0.5% MC as vehicles were grouped into a vehicle subset for the integrated HCD pool and used for the evaluation of VCGs. This consisted of seven studies with a total of 214 animals (107 males and 107 females, as detailed in Supplementary Table S2). Within this subset, the sample size was 15 animals per sex for six studies and 17 animals per sex for one study.

### 3.2 Assessing the consistency of toxicological interpretations after replacing CCGs with VCGs

To evaluate whether the VCG-based analysis reproduces the same toxicological interpretations as the CCG-based analysis, a reproducibility study was conducted on two legacy repeated-dose toxicity studies. The evaluation focused on body weight, clinicopathological parameters, and organ weight. In accordance with standard regulatory guidelines (e.g., OECD Test Guideline 408), sequential body weight measurements were evaluated independently as primary toxicological endpoints to assess test article-induced systemic toxicity. Along with body weight, organ weights and clinical pathology parameters were selected as the primary focuses of this evaluation, as they represent the core quantitative endpoints that provide objective, measurable data for assessing systemic toxicity in repeated-dose studies. Initially, the agreement in statistical significance (p ≤ 0.05) between CCGs and VCGs was compared. However, because clinical pathology parameters often exhibit high inherent biological variability that renders strict p-value reproduction unreliable, a category-based assessment considering the direction of change (increase/decrease) and percentage change in the data was conducted to effectively assess the biological relevance. Additionally, the toxicological interpretations derived from the VCG-based reanalyses were compared with meaningful changes identified by the SD in the original study reports.

#### 3.2.1 Body weight analysis

Supplementary Table S3 summarizes the results of VCG application on body weight changes measured at 7-day intervals. Although legacy study 1 showed no significant differences in body weight between the CCGs and treatment groups at any time point (14 non-significant time points in both sexes), the application of the VCGs yielded inconsistently significant differences in 86% (12 out of 14) of the measured time points in males. In contrast, the statistical analysis of body weight at all time points in females was consistently non-significant (14 of 14, 100%), thereby demonstrating perfect agreement in the statistical results between the CCGs and VCGs. In the legacy study 2, statistically significant differences were observed at five time points in males and seven in females. The comparison test with the VCGs reproduced identical statistical results at the same time points, thus confirming the perfect reproducibility of the VCG-based analysis (five out of five for males and seven out of seven for females). The mean body weights on day 1 were comparable with those of the CCGs, with differences maintained within a range of 5 g, except for females in legacy study 2, in which a relatively large difference of 24.5 g was observed between the two control groups (Supplementary Figure S3). However, differences in the growth rates between the CCGs and VCGs in males were observed across the measured time points in legacy study 1, which led to statistical significance at 86% of the time points (Supplementary Figure S4).

#### 3.2.2 Organ weight analysis

In legacy study 1, no statistically significant changes were observed in the organ weights in any treatment group for either sex (15 for males and 13 for females), and the application of VCGs produced similar results (Supplementary Table S4). Fourteen organ weights in males (93%) and all organ weights in females were classified as consistently non-significant, except for the salivary gland in males, which was classified as inconsistently significant. This confirmed the high reproducibility of VCG application.

In legacy study 2, statistically significant changes in organ weight were observed in three organs in males and one in females. When the weights were compared with those from the VCGs in males, 11 of the 12 organ weights with no statistical significance were confirmed to be consistently nonsignificant, and the brain weight was classified as inconsistently significant. In contrast, only one of three organ weights (33%) with statistical significance was classified as consistently significant, and the remaining two organ weights were classified as inconsistently non-significant. In females, consistently non-significant and significant organ weights were completely reproduced (Supplementary Table S4).

Overall, the statistical interpretations derived from the VCGs showed high agreement with those of the CCGs in both studies. This indicated that organ weight is a highly stable parameter when CCGs are replaced with VCGs.

#### 3.2.3 Analysis of clinical pathology parameters

For the clinical pathology parameters, VCGs were applied to 42 parameters for both sexes in each study and their reproducibility was evaluated. Perfectly replicating strict statistical significance thresholds (e.g., p ≤ 0.05) derived from CCGs is often difficult owing to limited sample sizes and inherent biological noise (Andaya et al., 2024; Gurjanov et al., 2024). In the current study, VCG analysis revealed this inconsistency (Supplementary Table S5).

In legacy study 1, the original analysis of the CCGs identified statistically significant differences in four parameters for males and seven parameters for females (Supplementary Table S5). However, the reanalysis using the VCGs demonstrated that only one parameter (mean corpuscular hemoglobin) among the four in males showed statistical significance (Supplementary Tables S5–S7). Some parameters, such as ALB and total protein (TP), which showed statistical differences in the CCG analysis, lost statistical significance in the VCG analysis. However, the category-based assessment demonstrated consistencies in the direction and magnitude of measurement changes in the parameters by attaining category 4 in the comparison between the VCG and T3 groups. This indicated their successful reproducibility in terms of biological relevance (Table 2). Ultimately, all evaluated parameters were classified into one of the reproducibility categories (1–4), thus achieving a 100% reproducibility rate based on biological relevance for both sexes (Table 3; Supplementary Table S10). Consequently, this category-based assessment allowed the VCG analysis to successfully replicate the toxicological interpretations of SDs. In this legacy study, the SD concluded that alterations in specific hematological parameters, including white blood cells (WBC), lymphocytes (LYM), platelets (PLAT), and neutrophils (NEUT) were associated with inflammatory responses in the gastric mucosa, whereas decreases in clinical chemistry markers (TP, ALB, and ALT) correlated with microstructural changes in the stomach and associated lymph nodes (pancreas and mesentery). The VCG-based reanalysis successfully reproduced these biomarker alterations, thereby maintaining the same direction of change and comparable percentage of changes (Tables 2, 4). These results confirmed that the fundamental toxicological profiles and target organ signals identified by SD remained robustly preserved when concurrent controls were substituted with VCGs.

**TABLE 2:** Category-based assessment of major clinical pathology findings based on the study director in male rats of legacy study 1.

Parameters	Dose	Result				Reproducibility Category
		CCG		VCG		
		Direction	Fold	Direction	Fold	
ALB	T3	Decrease	0.94*	Decrease	0.98	4
ALT	T3	Decrease	0.78*	Decrease	0.76*	2
LYM	T3	Increase	1.16	Increase	1.42	4
NEUT	T3	Increase	1.81	Increase	1.54	4
TP	T3	Decrease	0.93*	Decrease	0.96	4
WBC	T3	Increase	1.27	Increase	1.45*	4

* indicates statistical significance at p ≤ 0.05 compared with the respective control group. The reproducibility categories (1–5) were derived directly from the overall parameter evaluation detailed in Supplementary Table S10.

ALB, albumin; ALT, alanine aminotransferase; LYM, lymphocytes; NEUT, neutrophil; TP, total protein; WBC, white blood cell; CCG, concurrent control group; VCG, virtual control group.

**TABLE 3:** Biological reproducibility rates of clinical pathology parameters based on category-based assessment.

Category	Legacy study 1		Legacy study 2	
	Male	Female	Male	Female
	Parameters (n = 42)			
1	8 (19.1%)	6 (14.3%)	12 (28.6%)	10 (23.8%)
2	4 (9.5%)	5 (11.9%)	4 (9.5%)	1 (2.4%)
3	3 (7.1%)	4 (9.5%)	6 (14.3%)	5 (11.9%)
4	27 (64.3%)	27 (64.3%)	20 (47.6%)	26 (61.9%)
Total	42 (100%)	42 (100%)	42 (100%)	42 (100%)

**TABLE 4:** Category-based assessment of major clinical pathology findings based on the study director in female rats of legacy study 1.

Parameters	Dose	Result				Reproducibility Category
		CCG		VCG		
		Direction	Fold	Direction	Fold	
LYM	T2, T3	Increase	∼1.85	Increase	∼1.40	3
NEUT	T3	Increase	2.20	Increase	1.76*	4
PLAT	T3	Increase	1.12*	Increase	1.23*	4
WBC	T2, T3	Increase	∼1.90*	Increase	∼1.43	3

* indicates statistical significance at p ≤ 0.05 compared with the respective control group. The reproducibility categories (1–5) were derived directly from the overall parameter evaluation detailed in Supplementary Table S10.

LYM, lymphocytes; NEUT, neutrophil; PLAT, platelet; WBC, white blood cell; CCG, concurrent control group; VCG, virtual control group.

In the legacy study 2, the original CCG analysis identified statistically significant changes in 13 parameters in males and 10 in females (Supplementary Table S5). However, the reanalysis using the VCG demonstrated that only four parameters in males and four in females were consistently significant (Supplementary Tables S5, S8, S9). Replacing the CCG with the VCG yielded inconsistent statistical outcomes (e.g., inconsistently non-significant) for specific parameters, including AST and WBC.

However, all of these parameters were biologically consistent when a category-based assessment was applied. In particular, for key parameters, such as AST, increases at the mid and high doses (males, ≥T2) and WBC increases at the high dose (females, T3) lost their statistical significance in the VCG analysis (inconsistently non-significant). Nevertheless, the category-based assessment clearly demonstrated that the upward direction of change and fold changes (e.g., ∼1.39-fold for AST in males, and 1.15-fold for WBC in females) were consistently aligned with the CCG results (Tables 5, 6). Consequently, every evaluated parameter satisfied the criteria for reproducibility categories 1–4. This yielded a 100% reproducibility rate based on the biological relevance for both sexes (Table 3). Consistent with the findings of legacy study 1, this allowed the VCG analysis to successfully replicate the SD’s toxicological interpretations. For legacy study 2, the SD’s original report highlighted alterations in hepatocellular and inflammatory markers at the mid-dose level and above (≥T2). As specific hepatocellular markers, the SD noted clear increases in male ALT and AST levels at T2 and above. The VCG-based analysis consistently reproduced these trends, thereby maintaining the same direction of change and comparable fold-changes for both parameters (Table 5). Furthermore, as key indicators of the inflammatory response, SD emphasized increases in WBC, NEUT, LYM, MONO, and PLAT counts as major treatment-related changes. The VCG-based analysis successfully captured the upward direction of all these parameters. Although the fold changes for certain indicators showed minor variations, the fundamental inflammatory profile remained robustly consistent with the CCG-based results (Table 5).

**TABLE 5:** Category-based assessment of major clinical pathology findings based on the study director in male rats of legacy study 2.

Parameter	Dose	Result				Reproducibility Category
		CCG		VCG		
		Direction	Fold	Direction	Fold	
ALT	≥T2	Increase	∼2.72*	Increase	∼2.62*	1
AST	≥T2	Increase	∼1.56*	Increase	∼1.39	3
LYM	T3	Increase	1.18	Increase	1.14	1
MONO	T3	Increase	1.98*	Increase	1.67	4
NEUT	T3	Increase	1.53	Increase	1.58*	2
PLAT	T3	Increase	1.14	Increase	1.35*	3
WBC	T3	Increase	1.27	Increase	1.24	4

* indicates statistical significance at p ≤ 0.05 compared with the respective control group. The reproducibility categories (1–5) were derived directly from the overall parameter evaluation detailed in Supplementary Table S10.

ALT, alanine aminotransferase; AST, aspartate aminotransferase; LYM, lymphocytes; MONO, monocyte; NEUT, neutrophil; PLAT, platelet; WBC, white blood cell; CCG, concurrent control group; VCG, virtual control group.

**TABLE 6:** Category-based assessment of major clinical pathology findings based on the study director in female rats of legacy study two.

Parameter	Dose	Result				Reproducibility Category
		CCG		VCG		
		Direction	Fold	Direction	Fold	
LYM	T3	Increase	1.47*	Increase	1.15	3
MONO	T3	Increase	1.78	Increase	1.29	4
NEUT	T3	Increase	1.94*	Increase	1.25	4
PLAT	T3	Increase	1.16	Increase	1.49*	3
WBC	T3	Increase	1.54*	Increase	1.15	4

* indicates statistical significance at p ≤ 0.05 compared with the respective control group. The reproducibility categories (1–5) were derived directly from the overall parameter evaluation detailed in Supplementary Table S10.

ALT, alanine aminotransferase; AST, aspartate aminotransferase; LYM, lymphocytes; MONO, monocyte; NEUT, neutrophil; PLAT, platelet; WBC, white blood cell; CCG, concurrent control group; VCG, virtual control group.

## 4 Discussion

This study evaluates the feasibility of introducing VCG as part of the 3Rs initiative. The VCGs were generated based on the HCD of KIT and applied to two legacy studies to assess their reproducibility. Although endpoints, such as body weight and organ weight, demonstrated a highly consistent level of statistical significance between the CCG and VCG, the agreement in statistical significance (p-values) for clinical pathology parameters was relatively low at 25%–40%. However, by incorporating a category-based evaluation focused on the direction of change and fold changes and ultimately relying on the SD’s toxicological interpretation, this study proved that VCGs adequately replace CCGs to draw identical toxicological conclusions (e.g., target organ identification and inflammatory responses).

In this study, a Spearman correlation analysis confirmed a very high correlation (*ρ* > 0.9) in body weight, clinical pathology, and organ weight indicators among aqueous vehicles (saline, distilled water, and 0.5% MC), thereby allowing them to be consolidated into a single vehicle subset. Although pooling these vehicles might introduce a slight degree of statistical variance compared with that of a single vehicle, the fundamentally similar physicochemical properties of these aqueous formulations are expected to keep such variability minimal. The study findings demonstrate that this pooling strategy neither introduces severe statistical discrepancies nor alters the fundamental toxicological interpretations of SDs. Therefore, consolidating physicochemically similar vehicle groups is a practical and robust alternative for generating sufficiently powered VCGs and preventing severe HCD fragmentation highlighted in recent studies (Li et al., 2024; Palazzi et al., 2024).

While vehicle pooling mitigates data fragmentation, generating VCGs in the presence of inherent between-study variability alters the underlying statistical paradigm. Instead of testing the null hypothesis against a specific concurrent control mean (μ_H+1_) with limited within-study variance (σ_w_^2^), the VCG approach practically tests against an overall historical mean (μ) encompassing both within- and between-study variance (σ_w_^2^+σ_b_^2^). Relying strictly on conventional statistical significance under this widened variance risks acting merely as a data-driven assessment of the familywise error rate, potentially masking minor toxicological signals. Given this inherent between-study variation, implementing our proposed category-based biological relevance assessment becomes even more essential. By utilizing this evaluation framework, we effectively addressed these statistical limitations, demonstrating that all evaluated clinical pathology parameters were 100% biologically reproducible. The VCG data accurately captured key target organ toxicities (e.g., inflammatory responses and hepatocellular alterations) and fully supported the final toxicological interpretations. This aligns with recent reports demonstrating that major biologically relevant changes and NOAEL determinations remain consistent despite minor variations in exact statistical significance (Adedeji and Naor, 2025; Gurjanov et al., 2024; Lotfi et al., 2026). Furthermore, advanced Bayesian models have been highlighted as a future mathematical solution to account for this between-study variance (σ_b_^2^), improving estimation precision without violating the underlying statistical paradigm (Bowman et al., 2025; Li et al., 2024).

Furthermore, it is crucial to ensure that VCGs do not generate false-positive signals for parameters unaffected by the test article. In our study, a substantial proportion of endpoints lacking statistical significance and biological relevance in the original CCG analyses successfully retained their non-significant status under VCG analysis, including 92%–100% of unaffected organ weights and 57%–84% of unaffected clinical pathology parameters (Supplementary Tables S4, S5). This demonstrates that our VCG approach maintains high specificity at the endpoint level, effectively preventing the misinterpretation of negative findings as false positives, which aligns with recent findings in VCG methodologies evaluating specificity (Li et al., 2024; Wright et al., 2023).

Despite the overall success of the VCG approach in reproducing toxicological interpretations, it is important to acknowledge a limitation encountered in the current study. As observed in legacy study 1 (Section 3.2.1), a slightly lower growth rate in the VCG compared with the CCG over the 13-week period led to a high rate of statistical discrepancy (86%) in male body weights. This observation indicates that although matching initial (Day 1) body weights effectively controls for baseline age and developmental stage, it cannot fully eliminate the inherent between-study variations in time-course growth patterns over a subchronic period.

In nonclinical toxicology, the time-course increase in body weight (growth rate) over 13 weeks is not a static covariate but rather a dynamic, primary endpoint reflecting general systemic toxicity. While the difference in growth rates reduced the statistical reproducibility of body weight, we confirmed that it did not negatively impact the reproducibility of other toxicological endpoints.

To mitigate unmeasured temporal confounders, future methodologies must incorporate dynamic growth rate matching. While we evaluated 100% CCG replacement to test VCG limits, practical regulatory implementation will likely begin with a partial hybrid approach. Retaining a subset of ‘sentinel’ concurrent controls continuously assesses VCG-CCG comparability, detects unpredictable environmental confounders, and updates the historical database to prevent phenotypic drift (Grevot et al., 2023; Gurjanov et al., 2024; Lotfi et al., 2026). Expanding on this sentinel concept, future VCG generation can be approached through either proper statistical modeling or advanced artificial intelligence (AI) tools. While proper statistical models are essential to account for between-study variation and major covariates, generative AI algorithms, such as diffusion models, present a highly promising approach for dynamic matching. These AI models could utilize the growth rates of the sentinel CCGs as active inputs to synthesize or identify VCGs that perfectly match the dynamic profiles of the current study.

Although the current study successfully demonstrated the feasibility of VCGs using pooled aqueous vehicles and category-based assessments, further advancements are required to enhance their reliability and broad regulatory acceptance. As highlighted by recent studies (Andaya et al., 2024; Gurjanov et al., 2023; Palazzi et al., 2024), addressing inherent biological and site-specific baseline variability, such as varying anesthesia protocols and husbandry conditions, remains a critical challenge for VCG implementation. Furthermore, incorporating cross-validation with digitized histopathology (whole-slide imaging) is strongly recommended to quantitatively anchor the toxicological significance and resolve diagnostic discrepancies. Overall, embracing these emerging technological frameworks, along with the proposed pooling strategy, will improve the scientific credibility and practical utility of VCGs.

## 5 Conclusion

Although VCG research has primarily been pioneered and established by large-scale consortia in the European Union, this study critically validates its implementation in a distinct regulatory and laboratory context. Based on available studies, this study is the first to successfully implement and validate the VCG concept using region-specific HCD in the Republic of Korea. This pioneering work demonstrates the effective localization and validation of the VCG technology, thereby ensuring its robustness across different geographical and experimental environments.

Consistent with previous reports, the study results confirm that leveraging site-specific HCD is essential for controlling laboratory-specific variables and minimizing baseline variability. To address the practical challenge of limited data availability, the study proposes a strategic approach for expanding the HCD pool. By merging datasets with similar physiological profiles, specifically those using aqueous-based vehicles, based on a granular analysis of covariate impacts, the study demonstrated that the precision of VCG generation could be substantially enhanced without compromising data integrity.

Furthermore, successful VCG implementation requires a standardized methodological framework for validation rather than relying solely on statistical outcomes. To this end, this study introduces a category-based assessment framework as a robust methodology to evaluate VCG reproducibility. By systematically integrating statistical significance with a structured evaluation of biological relevance this methodology provides a rigorous framework to verify whether VCGs can successfully preserve the study director’s original toxicological interpretations.

Ultimately, this work serves as a practical framework for regulatory agencies and the pharmaceutical industry to develop future guidelines for animal safety assessments, thereby contributing to the global 3Rs principle.

## Data Availability

The data analyzed in this study is subject to the following licenses/restrictions: The datasets utilized in this research contain proprietary historical control data from legacy studies and are subject to strict non-disclosure and privacy restrictions. Due to these institutional and third-party confidentiality agreements, the raw datasets cannot be made publicly accessible. De-identified or summarized data may be shared upon reasonable request to the corresponding author, pending necessary administrative clearance. Requests to access these datasets should be directed to kimjw@kitox.re.kr.
