# Supplementary material for: A practical approach for regulatory acceptance of virtual control groups: vehicle pooling and biological relevance assessment
Source: Front Toxicol. 2026 Sep 4;8:1888158. doi: 10.3389/ftox.2026.1888158 (PMC13585411; doi:10.3389/ftox.2026.1888158)
Supplement: Supplementary file 1 [file Supplementaryfile1.docx]

Supplementary Material

# 1 Supplementary Figures and Tables

## 1.1 Supplementary Figures

**Supplementary Figure S1 (Continued)**

**Supplementary Figure S1 (Continued)**

**Supplementary Figure S1 (Continued)**

**Supplementary Figure S1 (Continued)**

**Supplementary Figure S1 (Continued)**

**Supplementary Figure S1 (Continued)**

**Supplementary Figure S1.** **Heatmap of Spearman correlation coefficients between administration vehicles for toxicological endpoints in male rats.** A high inter-vehicle correlation (median: 0.90, IQR: 0.85–0.99) demonstrates that the type of administration vehicle had limited impact on the baseline toxicological endpoints. Certain parameters were excluded for specific vehicle groups where those endpoints were not assessed in the original source studies. IQR, interquartile range; BW, body weight; MC, methylcellulose; DW, distilled water; CMC, carboxymethyl cellulose.

**Supplementary Figure S2 (Continued)**

**Supplementary Figure S2 (Continued)**

**Supplementary Figure S2 (Continued)**

**Supplementary Figure S2 (Continued)**

**Supplementary Figure S2 (Continued)**

**Supplementary Figure S2 (Continued)**

**Supplementary Figure S2 (Continued)**

**Supplementary Figure S2. Heatmap of Spearman correlation coefficients between administration vehicles for toxicological endpoints in female rats.** High inter-vehicle correlation (median: 0.93, IQR: 0.85–0.99) demonstrate that the type of administration vehicle has a limited impact on baseline toxicological endpoints. Certain parameters were excluded for specific vehicle groups where those endpoints were not assessed in the original source studies. IQR, interquartile range; BW, body weight; MC, methylcellulose; DW, distilled water; CMC, carboxymethyl cellulose.

**Supplementary Figure S3. Box-and-whisker plots of day 1 body weight in legacy study 1 and 2.** The plots illustrate the initial (day 1) body weight distribution of the concurrent control group (CCG) and generated virtual control group (VCG), thereby demonstrating the effectiveness of the preprocessing step in aligning the initial growth potential.

|  |  |
| --- | --- |
|  |  |

**Supplementary Figure S4. Body weight changes in legacy studies.** Body weight change curves for males and females over the 13-week study period. Overall, the growth trajectories of the virtual control groups (VCG) were parallel to those of the concurrent control groups (CCG). However, in males in legacy study 1, the VCG exhibited a slightly low growth rate over time, which contributed to the statistical significance observed in the treatment groups (Supplementary Table S3).

## 1.2 Supplementary Tables

Supplementary Table S1. Sample size and data sources of historical control data for vehicle effect analysis.

This table outlines the 14 legacy studies included in the initial historical control data pool. The table details the dosing duration, study year, administered vehicle types, and sample size of male and female rats for specific SEND domains to assess the impact of vehicle types on toxicological endpoints.

| Study^a^ | Dosing duration | Year  (Study start) | Vehicle | Endpoint^b^ | Sample size (Individuals, n) | |
| --- | --- | --- | --- | --- | --- | --- |
|  |  |  |  |  | Male | Female |
| A | 13 weeks | 2017 | Saline | BW | 15 | 15 |
|  |  |  |  | LB | 10 | 10 |
|  |  |  |  | OM | 10 | 10 |
| B | 13 weeks | 2017 | Saline | BW | 15 | 15 |
|  |  |  |  | OM | 10 | 10 |
| C | 13 weeks | 2017 | Saline | BW | 15 | 15 |
|  |  |  |  | LB | 10 | 10 |
|  |  |  |  | OM | 10 | 10 |
| D | 13 weeks | 2018 | 0.5% MC | BW | 15 | 15 |
|  |  |  |  | LB | 10 | 10 |
|  |  |  |  | OM | 10 | 10 |
| E | 26 weeks | 2019 | DW | BW | 10 | 10 |
| F | 13 weeks | 2019 | 0.5% MC | BW | 15 | 15 |
|  |  |  |  | LB | 10 | 10 |
|  |  |  |  | OM | 10 | 10 |
| G | 4 weeks | 2019 | Formulation^d^ | BW | 15 | 15 |
| H | 13 weeks | 2019 | 0.5% MC | BW | 15 | 15 |
|  |  |  |  | LB | 10 | 10 |
|  |  |  |  | OM | 10 | 10 |
| I | 26 weeks | 2019 | 0.5% MC | BW | 17 | 17 |
|  |  |  |  | OM | 12 | 12 |
| J | 4 weeks | 2020 | 0.5% CMC | BW | 15 | 15 |
| K | 13 weeks | 2020 | DW | BW | 15 | 15 |
|  |  |  |  | LB | 10 | 10 |
|  |  |  |  | OM | 10 | 10 |
| L | 26 weeks | 2020 | 0.5% CMC | BW | 20 | 20 |
|  |  |  |  | LB | 15 | 15 |
| M | 13 weeks | 2021 | 1% Tween 80 | BW | 15 | 15 |
|  |  |  |  | LB | 10 | 10 |
|  |  |  |  | OM | 10 | 10 |
| N | 26 weeks | 2021 | DW | BW | 17 | 17 |
|  |  |  |  | LB | 12 | 12 |
|  |  |  |  | OM | 12 | 12 |
| Total animals^c^ (Total: 428) | | | | | 214 | 214 |

^a^Anonymized identification

^b^ Standard for Exchange of Nonclinical Data (SEND) domains: BW, Body weight; LB, Laboratory; OM: Organ measurement

^c^Total number of animals was based on the BW domain, representing the actual unique individuals per study.

^d^Formulation refers to a specific vehicle provided by the study sponsor, the detailed composition of which is not disclosed.

MC, methylcellulose; DW, distilled water; CMC, carboxymethyl cellulose

**Supplementary Table S2. Sample size and data sources of the vehicle subset.**

This table provides detailed information on the legacy studies included in the specific physicochemical vehicle subset (saline, distilled water, and 0.5% methylcellulose) that exhibited high inter-vehicle correlations.

| Study^a^ | Dosing duration | Year  (Study start) | Vehicle | Endpoint^b^ | Sample size (Individuals, n) | |
| --- | --- | --- | --- | --- | --- | --- |
|  |  |  |  |  | Male | Female |
| A | 13 weeks | 2017 | Saline | BW | 15 | 15 |
|  |  |  |  | LB | 10 | 10 |
|  |  |  |  | OM | 10 | 10 |
| C | 13 weeks | 2017 | Saline | BW | 15 | 15 |
|  |  |  |  | LB | 10 | 10 |
|  |  |  |  | OM | 10 | 10 |
| D | 13 weeks | 2018 | 0.5% MC | BW | 15 | 15 |
|  |  |  |  | LB | 10 | 10 |
|  |  |  |  | OM | 10 | 10 |
| F | 13 weeks | 2019 | 0.5% MC | BW | 15 | 15 |
|  |  |  |  | LB | 10 | 10 |
|  |  |  |  | OM | 10 | 10 |
| H | 13 weeks | 2019 | 0.5% MC | BW | 15 | 15 |
|  |  |  |  | LB | 10 | 10 |
|  |  |  |  | OM | 10 | 10 |
| K | 13 weeks | 2020 | DW | BW | 15 | 15 |
|  |  |  |  | LB | 10 | 10 |
|  |  |  |  | OM | 10 | 10 |
| N | 26 weeks^c^ | 2021 | DW | BW | 17 | 17 |
|  |  |  |  | LB | 12 | 12 |

^a^Anonymized identification

^b^Standard for Exchange of Nonclinical Data (SEND) domains: BW, Body weight; LB, Laboratory test results; OM, Organ measurements

^c^For study N (a 26-week study), only the data measured and observed at the 13-week point were extracted and included in the subset to strictly match the duration of the legacy studies.

**Supplementary Table S3. Comparison of statistical significance between CCG and VCG in body weight data.**

This table summarizes the reproducibility of the statistical significance of body weight changes across legacy studies 1 and 2.

| Control | Category | Legacy study 1 (n = 14) | | Legacy study 2 (n = 14) | |
| --- | --- | --- | --- | --- | --- |
|  |  | Male | Female | Male | Female |
| CCG | Significantly different n (p ≤ 0.05) | 0 | 0 | 5 | 7 |
|  | Not-significantly different n (p ≤ 0.05) | 14 | 14 | 9 | 7 |
| VCG (Reproducibility with CCG results) | Consistently significant | 0/0  (NA) | 0/0  (NA) | 5/5  (100%) | 7/7  (100%) |
|  | Consistently non-significant | 2/14  (14%) | 14/14  (100%) | 8/9  (89%) | 5/7  (71%) |
|  | Inconsistently significant | 12/14  (86%) | 0/14  (0%) | 1/9  (11%) | 2/7  (29%) |
|  | Inconsistently non-significant | 0/0  (NA) | 0/0  (NA) | 0/5  (0%) | 0/7  (0%) |
|  | Inversely significant | 0/0  (NA) | 0/0  (NA) | 0/5  (0%) | 0/7  (0%) |

n, number of body weight time points evaluated; NA, not applicable; CCG, concurrent control group; VCG, virtual control group.

**Supplementary Table S4. Comparison of statistical significance between CCG and VCG in organ weight data.**

This table presents the statistically significant agreements for absolute organ weights. The high rate of consistently non-significant and significant outcomes indicated that organ weight is a highly stable parameter for VCG application.

| Control | Category | Legacy study 1 | | Legacy study 2 | |
| --- | --- | --- | --- | --- | --- |
|  |  | Male  (n = 15) | Female  (n = 13) | Male  (n = 15) | Female  (n = 13) |
| CCG | Significantly different n (p ≤ 0.05) | 0 | 0 | 3 | 1 |
|  | Not-significantly different n (p ≤ 0.05) | 15 | 13 | 12 | 12 |
| VCG (Reproducibility with CCG results) | Consistently significant | 0/0  (NA) | 0/0  (NA) | 1/3  (33%) | 1/1  (100%) |
|  | Consistently non-significant | 14/15  (93%) | 13/13  (100%) | 11/12  (92%) | 12/12  (100%) |
|  | Inconsistently significant | 1/15  (7%) | 0/13  (0%) | 1/12  (8%) | 0/12  (0%) |
|  | Inconsistently non-significant | 0/0  (NA) | 0/0  (NA) | 2^†^/3  (67%) | 0/1  (0%) |
|  | Inversely significant | 0/0  (NA) | 0/0  (NA) | 0/3  (0%) | 0/1  (0%) |

n, number of organ weight parameters evaluated; NA, not applicable; CCG, concurrent control group; VCG, virtual control group.

^†^For males in legacy study 2, two findings that were initially significant under CCG became non-significant under VCG analysis (inconsistently non-significant). However, the direction and magnitude of changes in these organs were comparable with those observed in the CCG, which lacked statistical significance.

**Supplementary Table S5. Statistical comparison of clinical pathology results between CCG and VCG.**

This table provides a detailed breakdown of statistical agreement (p-values) for 42 clinical pathology parameters. The relatively low rate of exact p-value reproduction highlights the limitations of relying solely on strict statistical thresholds in clinical pathological evaluations.

| Control | | Category | Legacy study 1 | | Legacy study 2 | |
| --- | --- | --- | --- | --- | --- | --- |
|  |  |  | Male  (n = 42) | Female  (n = 42) | Male  (n = 42) | Female  (n = 42) |
| CCG | Significantly different n (p ≤ 0.05) | | 4 | 7 | 13 | 10 |
|  | Not-significantly different n (p ≤ 0.05) | | 38 | 35 | 29 | 32 |
| VCG (Reproducibility with CCG results) | Consistently significant | | 1/4  (25%) | 0/7  (0%) | 4/13  (31%) | 4/10  (40%) |
|  | Consistently non-significant | | 31/38  (82%) | 20/35  (57%) | 22/29  (76%) | 27/32  (84%) |
|  | Inconsistently significant | | 9/38  (24%) | 16/35  (46%) | 9/29  (31%) | 7/32  (22%) |
|  | Inconsistently non-significant | | 2/4  (50%) | 5/7  (71%) | 7/13  (54%) | 4/10  (40%) |
|  | Inversely significant | | 0/4  (0%) | 1/7  (14%) | 0/13  (0%) | 0/10  (0%) |

n, number of clinical pathology parameters evaluated; CCG, concurrent control group; VCG, virtual control group.

**Supplementary Table S6. Comparison of percent changes in clinical pathology parameters of male rats in legacy study 1, CCG, and VCG.**

This table presents detailed directional changes, magnitude (percent change), and statistical significance of individual clinical pathology parameters in male rats of legacy study 1.

|  | Group | | | | | | | | | | | |
| --- | --- | --- | --- | --- | --- | --- | --- | --- | --- | --- | --- | --- |
|  | **CCG vs. T1** | | **CCG vs. T2** | | **CCG vs. T3** | | **VCG vs. T1** | | **VCG vs. T2** | | **VCG vs. T3** | |
| Parameter | (%) | Change | (%) | Change | (%) | Change | (%) | Change | (%) | Change | (%) | Change |
| ALB | −3.05 | ↓ | −1.21 | ↓ | −6.26 | ↓* | 1.10 | ↑ | 3.01 | ↑ | −2.25 | ↓ |
| ALBGLOB | −1.66 | ↓ | 0.63 | ↑ | 2.92 | ↑ | -0.46 | ↓ | 1.85 | ↑ | 4.17 | ↑ |
| ALP | −7.52 | ↓ | −11.38 | ↓ | −18.20 | ↓ | 4.54 | ↑ | 0.18 | ↑ | −7.53 | ↓ |
| ALT | −15.57 | ↓ | −15.51 | ↓ | −21.80 | ↓* | −18.23 | ↓* | −18.17 | ↓* | −24.26 | ↓* |
| APTT | −5.17 | ↓ | −4.80 | ↓ | −9.64 | ↓ | 4.61 | ↑ | 5.02 | ↑ | −0.33 | ↓ |
| AST | -4.13 | ↓ | 3.73 | ↑ | −2.65 | ↓ | −0.11 | ↓ | 8.08 | ↑ | 1.43 | ↑ |
| BASO | 15.38 | ↑ | 34.62 | ↑↑ | 53.85 | ↑↑↑ | 17.65 | ↑ | 37.25 | ↑↑ | 56.86 | ↑↑↑ |
| BASOLE | 25.00 | ↑ | 25.00 | ↑ | 20.00 | ↑ | 11.11 | ↑ | 11.11 | ↑ | 6.67 | ↑ |
| BILI | 14.93 | ↑ | 18.89 | ↑ | −0.92 | ↓ | 19.18 | ↑ | 23.29 | ↑ | 2.74 | ↑ |
| CA | 0.11 | ↑ | −0.62 | ↓ | −2.16 | ↓ | 3.17 | ↑ | 2.42 | ↑ | 0.83 | ↑ |
| CHOL | −7.04 | ↓ | −14.08 | ↓ | −25.63 | ↓↓ | −2.17 | ↓ | −9.57 | ↓ | −21.73 | ↓ |
| CK | 0.17 | ↑ | 11.55 | ↑ | 12.69 | ↑ | 1.80 | ↑ | 13.36 | ↑ | 14.53 | ↑ |
| CL | 1.00 | ↑ | 1.30 | ↑ | 1.80 | ↑ | −1.85 | ↓* | −1.55 | ↓ | −1.07 | ↓ |
| CREAT | −1.16 | ↓ | 4.65 | ↑ | 2.71 | ↑ | 2.62 | ↑ | 8.65 | ↑ | 6.64 | ↑ |
| EOS | 14.29 | ↑ | 0.00 | - | 0.00 | - | 24.03 | ↑ | 8.53 | ↑ | 8.53 | ↑ |
| EOSLE | 13.04 | ↑ | −11.30 | ↓ | −19.13 | ↓ | 2.36 | ↑ | −19.69 | ↓ | −26.77 | ↓↓ |
| GGT | 16.67 | ↑ | 88.89 | ↑↑↑ | −33.33 | ↓↓ | −69.48 | ↓↓↓ | −50.58 | ↓↓↓ | −82.56 | ↓↓↓* |
| GLUC | −3.31 | ↓ | −8.68 | ↓ | −5.39 | ↓ | 0.63 | ↑ | −4.97 | ↓ | −1.54 | ↓ |
| HCT | −0.02 | ↓ | 1.22 | ↑ | −2.21 | ↓ | 4.04 | ↑ | 5.34 | ↑ | 1.76 | ↑ |
| HGB | 3.04 | ↑ | 3.72 | ↑ | −1.86 | ↓ | 5.67 | ↑ | 6.37 | ↑* | 0.64 | ↑ |
| K | 0.95 | ↑ | −4.90 | ↓ | 3.24 | ↑ | −2.95 | ↓ | −8.57 | ↓ | −0.75 | ↓ |
| LGLUCLE | 6.38 | ↑ | 38.30 | ↑↑ | 10.64 | ↑ | −7.41 | ↓ | 20.37 | ↑ | −3.70 | ↓ |
| LGUNSCE | −0.83 | ↓ | 48.76 | ↑↑ | 40.50 | ↑↑ | 3.45 | ↑ | 55.17 | ↑↑↑ | 46.55 | ↑↑ |
| LYM | −0.21 | ↓ | 4.78 | ↑ | 16.36 | ↑ | 21.59 | ↑ | 27.67 | ↑↑ | 41.77 | ↑↑ |
| LYMLE | 3.21 | ↑ | 0.86 | ↑ | −6.44 | ↓ | 9.04 | ↑* | 6.56 | ↑ | −1.15 | ↓ |
| MCH | 5.91 | ↑* | 5.21 | ↑* | 2.28 | ↑ | 5.48 | ↑* | 4.78 | ↑* | 1.86 | ↑ |
| MCHC | 2.86 | ↑ | 2.39 | ↑ | 0.31 | ↑ | 1.32 | ↑ | 0.85 | ↑ | −1.19 | ↓ |
| MCV | 2.96 | ↑ | 2.79 | ↑ | 2.01 | ↑ | 3.97 | ↑* | 3.80 | ↑* | 3.01 | ↑ |
| MONO | 1.35 | ↑ | 26.13 | ↑↑ | 59.91 | ↑↑↑ | 13.07 | ↑ | 40.70 | ↑↑ | 78.39 | ↑↑↑* |
| MONOLE | 2.27 | ↑ | 17.33 | ↑ | 23.58 | ↑ | 0.56 | ↑ | 15.36 | ↑ | 21.51 | ↑ |
| NEUT | −14.55 | ↓ | 1.04 | ↑ | 81.29 | ↑↑↑ | −27.20 | ↓↓ | −13.92 | ↓ | 54.45 | ↑↑↑ |
| NEUTLE | −19.08 | ↓ | −10.66 | ↓ | 28.16 | ↑↑ | −35.62 | ↓↓* | −28.92 | ↓↓* | 1.96 | ↑ |
| PHOS | 1.38 | ↑ | −1.83 | ↓ | 2.13 | ↑ | 5.83 | ↑ | 2.48 | ↑ | 6.61 | ↑ |
| PLAT | −1.89 | ↓ | 1.00 | ↑ | 5.14 | ↑ | − 3.74 | ↓ | −0.91 | ↓ | 3.15 | ↑ |
| PROT | −2.46 | ↓ | −1.44 | ↓ | −7.26 | ↓* | 1.40 | ↑ | 2.46 | ↑ | −3.59 | ↓ |
| PT | −3.58 | ↓ | −4.60 | ↓ | −1.47 | ↓ | −2.61 | ↓ | −3.64 | ↓ | −0.48 | ↓ |
| RBC | −2.55 | ↓ | −1.39 | ↓ | −3.93 | ↓ | 0.47 | ↑ | 1.67 | ↑ | −0.95 | ↓ |
| RETI | 3.21 | ↑ | −1.55 | ↓ | 1.02 | ↑ | −1.83 | ↓ | −6.36 | ↓ | −3.92 | ↓ |
| RETIRBC | 6.11 | ↑ | 0.05 | ↑ | 8.64 | ↑ | −1.98 | ↓ | −7.58 | ↓ | 0.35 | ↑ |
| SODIUM | −0.07 | ↓ | 0.34 | ↑ | −0.14 | ↓ | 1.24 | ↑ | 1.66 | ↑ | 1.17 | ↑ |
| TRIG | 37.58 | ↑↑ | −5.24 | ↓ | 18.26 | ↑ | 80.97 | ↑↑↑ | 24.65 | ↑ | 55.56 | ↑↑↑ |
| WBC | −1.70 | ↓ | 5.49 | ↑ | 26.99 | ↑↑ | 11.97 | ↑ | 20.16 | ↑ | 44.65 | ↑↑* |

* Statistically significant difference compared to the control group (p < 0.05)

↑ Increase; ↓ Decrease; - No change. The number of arrows indicates the relative magnitude of percent change compared with that of the control group (1 arrow: <25%, 2 arrows: 25 ≤ 50%, 3 arrows: 50 ≤ 100%, 4 arrows: ≥100%).

Note: Clinical pathology parameters are presented using Standard for Exchange of Nonclinical Data (SEND) terminology to ensure data interoperability.

CCG, concurrent control group; VCG, virtual control group;

Abbreviations: ALB, albumin; ALBGLOB, albumin/globulin ratio; ALP, alkaline phosphatase; ALT, alanine aminotransferase; APTT, activated partial thromboplastin time; AST, aspartate aminotransferase; BASO, basophils (absolute); BASOLE, basophils/leukocytes (relative); BILI, total bilirubin; CA, calcium; CHOL, total cholesterol; CK, creatine kinase; CL, chloride; CREAT, creatinine; EOS, eosinophils (absolute); EOSLE, eosinophils/leukocytes (relative); GGT, gamma-glutamyl transferase; GLUC, glucose; HCT, hematocrit; HGB, hemoglobin; K, potassium; LGLUCLE, large unstained cells/leukocytes (relative); LGUNSCE, large unstained cells (absolute); LYM, lymphocytes (absolute); LYMLE, lymphocytes/leukocytes (relative); MCH, mean corpuscular hemoglobin; MCHC, mean corpuscular hemoglobin concentration; MCV, mean corpuscular volume; MONO, monocytes (absolute); MONOLE, monocytes/leukocytes (relative); NEUT, neutrophils (absolute); NEUTLE, neutrophils/leukocytes (relative); PHOS, inorganic phosphorus; PLAT, platelets; PROT, total protein; PT, prothrombin time; RBC, red blood cells; RETI, reticulocytes (absolute); RETIRBC, reticulocytes/erythrocytes (relative); SODIUM, sodium; TRIG, triglycerides; WBC, white blood cells.

**Supplementary Table S7. Comparison of percent changes in clinical pathology parameters of female rats in legacy study 1, CCG, and VCG.**

This table presents detailed directional changes, magnitude (percent change), and statistical significance of individual clinical pathology parameters in female rats of the legacy study 1.

|  |  | |  |  | |  | | Group | |  | |  | |
| --- | --- | --- | --- | --- | --- | --- | --- | --- | --- | --- | --- | --- | --- |
|  | **CCG vs. T1** | | | **CCG vs. T2** | | **CCG vs. T3** | | **VCG vs. T1** | | **VCG vs. T2** | | **VCG vs. T3** | |
| Parameter | (%) | Change | | (%) | Change | (%) | Change | (%) | Change | (%) | Change | (%) | Change |
| ALB | 2.41 | ↑ | | 2.49 | ↑ | 0.23 | ↑ | −2.26 | ↓ | −2.18 | ↓* | −4.33 | ↓* |
| ALBGLOB | 6.06 | ↑ | | 11.01 | ↑* | 6.46 | ↑ | −10.16 | ↓ | −5.96 | ↓ | −9.82 | ↓ |
| ALP | −24.25 | ↓ | | −18.45 | ↓ | −12.88 | ↓ | −2.44 | ↓ | 5.02 | ↑ | 12.19 | ↑ |
| ALT | 6.62 | ↑ | | −12.98 | ↓ | −19.20 | ↓ | −29.07 | ↓↓ | −42.11 | ↓↓* | −46.24 | ↓↓* |
| APTT | −5.48 | ↓ | | −2.59 | ↓ | −0.62 | ↓ | 5.54 | ↑* | 8.77 | ↑ | 10.97 | ↑ |
| AST | −8.86 | ↓ | | −7.28 | ↓ | −12.63 | ↓ | −20.54 | ↓ | −19.16 | ↓ | −23.83 | ↓ |
| BASO | 6.45 | ↑ | | 45.16 | ↑↑ | 51.61 | ↑↑↑ | 0.00 | - | 36.36 | ↑↑ | 42.42 | ↑↑* |
| BASOLE | −23.64 | ↓ | | −3.64 | ↓ | −20.00 | ↓ | −4.55 | ↓ | 20.45 | ↑ | 0.00 | - |
| BILI | 18.32 | ↑ | | 21.23 | ↑ | 16.83 | ↑ | 9.29 | ↑ | 11.98 | ↑ | 7.92 | ↑ |
| CA | 0.38 | ↑ | | 0.56 | ↑ | −0.23 | ↓ | 2.14 | ↑ | 2.32 | ↑ | 1.51 | ↑ |
| CHOL | 16.67 | ↑ | | −1.17 | ↓ | 13.41 | ↑ | 7.56 | ↑ | −8.88 | ↓ | 4.56 | ↑* |
| CK | −28.49 | ↓↓ | | −16.29 | ↓ | −19.90 | ↓ | −23.15 | ↓ | −10.03 | ↓ | −13.91 | ↓ |
| CL | 0.39 | ↑ | | −0.78 | ↓ | 1.76 | ↑* | −2.56 | ↓ | −3.69 | ↓ | −1.23 | ↓* |
| CREAT | -8.30 | ↓ | | −6.01 | ↓ | −12.16 | ↓* | 7.82 | ↑ | 10.51 | ↑ | 3.28 | ↑ |
| EOS | 30.56 | ↑↑ | | 13.89 | ↑ | 59.72 | ↑↑↑* | 2.17 | ↑ | −10.87 | ↓ | 25.00 | ↑ |
| EOSLE | −7.14 | ↓ | | −20.71 | ↓ | −17.14 | ↓ | −1.14 | ↓ | −15.59 | ↓ | −11.79 | ↓* |
| GGT | −12.52 | ↓ | | −32.70 | ↓↓ | −46.85 | ↓↓ | 43.53 | ↑↑ | 10.43 | ↑ | −12.78 | ↓ |
| GLUC | −6.18 | ↓ | | −4.97 | ↓ | −13.52 | ↓ | −1.47 | ↓ | −0.20 | ↓ | −9.18 | ↓ |
| HCT | −2.48 | ↓ | | −0.20 | ↓ | 0.57 | ↑ | 6.37 | ↑ | 8.86 | ↑ | 9.70 | ↑ |
| HGB | −1.81 | ↓ | | −0.06 | ↓ | 0.78 | ↑ | 4.15 | ↑ | 6.01 | ↑* | 6.90 | ↑* |
| K | −6.13 | ↓ | | 3.56 | ↑ | −1.66 | ↓ | 10.99 | ↑ | 22.45 | ↑* | 16.28 | ↑ |
| LGLUCLE | 8.08 | ↑ | | 25.25 | ↑↑ | 13.13 | ↑ | 16.94 | ↑ | 35.52 | ↑↑* | 22.40 | ↑ |
| LGUNSCE | 58.18 | ↑↑↑ | | 76.36 | ↑↑↑ | 118.18 | ↑↑↑↑* | 26.09 | ↑↑ | 40.58 | ↑↑ | 73.91 | ↑↑↑ |
| LYM | 42.28 | ↑↑ | | 53.07 | ↑↑↑ | 84.99 | ↑↑↑ | 7.45 | ↑ | 15.60 | ↑ | 39.71 | ↑↑ |
| LYMLE | 0.66 | ↑ | | 5.23 | ↑ | −2.50 | ↓ | 0.25 | ↑ | 4.80 | ↑ | −2.90 | ↓ |
| MCH | 1.23 | ↑ | | −0.16 | ↓ | 0.80 | ↑ | 1.02 | ↑ | −0.37 | ↓* | 0.59 | ↑* |
| MCHC | 0.64 | ↑ | | 0.09 | ↑ | 0.15 | ↑ | −2.21 | ↓ | −2.75 | ↓ | −2.69 | ↓* |
| MCV | 0.61 | ↑ | | −0.16 | ↓ | 0.67 | ↑ | 3.12 | ↑ | 2.33 | ↑ | 3.18 | ↑ |
| MONO | 23.96 | ↑ | | 42.19 | ↑↑ | 94.79 | ↑↑↑ | −8.11 | ↓ | 5.41 | ↑ | 44.40 | ↑↑ |
| MONOLE | −13.31 | ↓ | | −1.70 | ↓ | −5.95 | ↓ | −15.00 | ↓ | −3.61 | ↓ | −7.78 | ↓ |
| NEUT | 30.73 | ↑↑ | | 0.00 | - | 119.57 | ↑↑↑↑ | 4.91 | ↑ | −19.75 | ↓ | 76.20 | ↑↑↑* |
| NEUTLE | 0.46 | ↑ | | −26.43 | ↓↓ | 15.23 | ↑ | 1.40 | ↑ | −25.75 | ↓↓ | 16.30 | ↑ |
| PHOS | −6.56 | ↓ | | −0.74 | ↓ | 0.48 | ↑ | 2.90 | ↑ | 9.32 | ↑ | 10.66 | ↑ |
| PLAT | −4.47 | ↓ | | −3.54 | ↓ | 11.92 | ↑* | 5.22 | ↑* | 6.25 | ↑* | 23.28 | ↑* |
| PROT | 0.17 | ↑ | | −1.36 | ↓ | −2.10 | ↓ | 0.81 | ↑ | −0.74 | ↓* | −1.48 | ↓* |
| PT | −0.88 | ↓ | | −2.11 | ↓ | −0.68 | ↓ | 6.42 | ↑ | 5.11 | ↑* | 6.64 | ↑* |
| RBC | −3.17 | ↓ | | −0.06 | ↓ | −0.06 | ↓ | 3.33 | ↑ | 6.66 | ↑ | 6.66 | ↑ |
| RETI | 0.79 | ↑ | | 5.25 | ↑ | 5.14 | ↑ | −8.50 | ↓ | −4.45 | ↓ | −4.55 | ↓ |
| RETIRBC | 3.87 | ↑ | | 5.53 | ↑ | 4.92 | ↑ | −12.34 | ↓ | −10.94 | ↓ | −11.45 | ↓ |
| SODIUM | 0.41 | ↑ | | −0.21 | ↓ | −0.21 | ↓ | −0.07 | ↓ | −0.68 | ↓* | −0.68 | ↓ |
| TRIG | −5.75 | ↓ | | −3.28 | ↓ | 4.01 | ↑ | −18.94 | ↓ | −16.82 | ↓ | −10.55 | ↓ |
| WBC | 39.68 | ↑↑ | | 44.29 | ↑↑* | 90.30 | ↑↑↑* | 5.07 | ↑ | 8.54 | ↑ | 43.15 | ↑↑ |

* Statistically significant difference compared to the control group (p < 0.05)

↑ Increase; ↓ Decrease; - No change. The number of arrows indicates the relative magnitude of percent change compared with that of the control group (1 arrow: <25%, 2 arrows: 25 ≤ 50%, 3 arrows: 50 ≤ 100%, 4 arrows: ≥100%).

Note: Clinical pathology parameters are presented using Standard for Exchange of Nonclinical Data (SEND) terminology to ensure data interoperability.

CCG, concurrent control group; VCG, virtual control group;

Abbreviations: ALB, albumin; ALBGLOB, albumin/globulin ratio; ALP, alkaline phosphatase; ALT, alanine aminotransferase; APTT, activated partial thromboplastin time; AST, aspartate aminotransferase; BASO, basophils (absolute); BASOLE, basophils/leukocytes (relative); BILI, total bilirubin; CA, calcium; CHOL, total cholesterol; CK, creatine kinase; CL, chloride; CREAT, creatinine; EOS, eosinophils (absolute); EOSLE, eosinophils/leukocytes (relative); GGT, gamma-glutamyl transferase; GLUC, glucose; HCT, hematocrit; HGB, hemoglobin; K, potassium; LGLUCLE, large unstained cells/leukocytes (relative); LGUNSCE, large unstained cells (absolute); LYM, lymphocytes (absolute); LYMLE, lymphocytes/leukocytes (relative); MCH, mean corpuscular hemoglobin; MCHC, mean corpuscular hemoglobin concentration; MCV, mean corpuscular volume; MONO, monocytes (absolute); MONOLE, monocytes/leukocytes (relative); NEUT, neutrophils (absolute); NEUTLE, neutrophils/leukocytes (relative); PHOS, inorganic phosphorus; PLAT, platelets; PROT, total protein; PT, prothrombin time; RBC, red blood cells; RETI, reticulocytes (absolute); RETIRBC, reticulocytes/erythrocytes (relative); SODIUM, sodium; TRIG, triglycerides; WBC, white blood cells.

**Supplementary Table S8. Comparison of percent changes in clinical pathology parameters of male rats in legacy study 2, CCG, and VCG.**

The table presents detailed directional changes, magnitude (percent change), and statistical significance of individual clinical pathology parameters in male rats of legacy study 2.

|  | Group | | | | | | | | | | | |
| --- | --- | --- | --- | --- | --- | --- | --- | --- | --- | --- | --- | --- |
|  | **CCG vs. T1** | | **CCG vs. T2** | | **CCG vs. T3** | | **VCG vs.T1** | | **VCG vs. T2** | | **VCG vs. T3** | |
| Parameter | (%) | Change | (%) | Change | (%) | Change | (%) | Change | (%) | Change | (%) | Change |
| ALB | 0.68 | ↑ | −2.49 | ↓ | −4.54 | ↓ | −5.40 | ↓ | −8.38 | ↓* | −10.30 | ↓* |
| ALBGLOB | 1.43 | ↑ | 8.26 | ↑* | 12.01 | ↑* | −3.48 | ↓ | 3.03 | ↑ | 6.59 | ↑ |
| ALP | −2.10 | ↓ | −1.09 | ↓ | −0.89 | ↓ | −6.48 | ↓ | −5.52 | ↓ | −5.33 | ↓ |
| ALT | 14.60 | ↑ | 63.32 | ↑↑↑* | 171.69 | ↑↑↑↑* | 10.55 | ↑ | 57.55 | ↑↑↑* | 162.09 | ↑↑↑↑* |
| APTT | −4.22 | ↓ | −14.16 | ↓* | −11.44 | ↓* | 8.38 | ↑ | −2.87 | ↓ | 0.21 | ↑ |
| AST | −8.09 | ↓ | 21.88 | ↑ | 55.85 | ↑↑↑* | −18.07 | ↓ | 8.65 | ↑ | 38.93 | ↑↑ |
| BASO | −9.62 | ↓ | 3.85 | ↑ | 48.08 | ↑↑ | −4.08 | ↓ | 0.20 | ↑ | 57.14 | ↑↑↑* |
| BASOLE | −2.13 | ↓ | −2.13 | ↓ | 14.89 | ↑ | 15.00 | ↑ | 15.00 | ↑ | 35.00 | ↑↑ |
| BILI | −0.56 | ↓ | 9.05 | ↑ | 16.91 | ↑ | 0.11 | ↑ | 9.78 | ↑ | 17.70 | ↑ |
| CA | 1.89 | ↑ | 0.93 | ↑ | −0.92 | ↓ | −1.25 | ↓ | −2.18 | ↓ | −3.98 | ↓ |
| CHOL | −1.33 | ↓ | −20.42 | ↓ | −28.91 | ↓↓* | −0.73 | ↓ | −19.95 | ↓ | −28.49 | ↓↓* |
| CK | −29.95 | ↓↓ | −13.00 | ↓ | −12.16 | ↓ | −29.42 | ↓↓ | −12.34 | ↓ | −11.49 | ↓ |
| CL | 0.49 | ↑ | 1.57 | ↑ | 3.73 | ↑* | 0.05 | ↑ | 1.12 | ↑ | 3.27 | ↑* |
| CREAT | 3.07 | ↑ | −4.92 | ↓ | −6.97 | ↓ | 0.50 | ↑ | −7.29 | ↓ | −9.29 | ↓ |
| EOS | −14.02 | ↓ | 0.00 | - | −7.48 | ↓ | −36.33 | ↓↓ | −25.95 | ↓↓ | −31.49 | ↓↓ |
| EOSLE | −12.87 | ↓ | −2.97 | ↓ | −29.70 | ↓↓ | −34.33 | ↓↓ | −26.87 | ↓↓ | −47.01 | ↓↓* |
| GGT | −24.68 | ↓ | −74.89 | ↓↓↓* | −48.48 | ↓↓ | −21.00 | ↓ | −73.67 | ↓↓↓ | −45.97 | ↓↓ |
| GLUC | 10.42 | ↑ | 6.35 | ↑ | 3.91 | ↑ | 6.95 | ↑ | 3.00 | ↑ | 0.64 | ↑ |
| HCT | −1.26 | ↓ | −3.72 | ↓ | −2.82 | ↓ | 2.04 | ↑ | −0.51 | ↓ | 0.43 | ↑ |
| HGB | −1.32 | ↓ | −4.29 | ↓ | −5.30 | ↓ | −2.19 | ↓ | −5.13 | ↓* | −6.13 | ↓* |
| K | −4.06 | ↓ | 8 −65 | ↑ | 0.47 | ↑ | 13.39 | ↑ | 28.41 | ↑↑* | 18.74 | ↑ |
| LGLUCLE | 6.25 | ↑ | 24.11 | ↑ | 7.14 | ↑ | 30.77 | ↑↑ | 52.75 | ↑↑↑ | 31.87 | ↑↑ |
| LGUNSCE | 3.97 | ↑ | 30.95 | ↑↑ | 35.71 | ↑↑ | 31.66 | ↑↑ | 65.83 | ↑↑↑ | 71.86 | ↑↑↑ |
| LYM | −8.73 | ↓ | −3.05 | ↓ | 18.41 | ↑ | −11.98 | ↓ | −6.49 | ↓ | 14.20 | ↑ |
| LYMLE | −2.71 | ↓ | −5.20 | ↓ | −6.41 | ↓ | −1.71 | ↓ | −4.23 | ↓ | −5.45 | ↓ |
| MCH | 1.85 | ↑ | −0.66 | ↓ | −4.71 | ↓* | −1.70 | ↓ | −4.11 | ↓ | −8.02 | ↓* |
| MCHC | −0.16 | ↓ | −0.67 | ↓ | −2.43 | ↓* | −4.37 | ↓ | −4.86 | ↓ | −6.55 | ↓ |
| MCV | 2.12 | ↑ | 0.07 | ↑ | −2.21 | ↓ | 2.86 | ↑ | 0.80 | ↑ | −1.51 | ↓ |
| MONO | 2.64 | ↑ | 14.08 | ↑ | 98.24 | ↑↑↑* | −14.22 | ↓ | −4.66 | ↓ | 65.69 | ↑↑↑ |
| MONOLE | 7.26 | ↑ | 12.54 | ↑ | 49.83 | ↑↑* | −9.22 | ↓ | −4.75 | ↓ | 26.82 | ↑↑ |
| NEUT | 6.28 | ↑ | 15.19 | ↑ | 53.32 | ↑↑↑ | 9.36 | ↑ | 18.53 | ↑ | 57.77 | ↑↑↑* |
| NEUTLE | 10.87 | ↑ | 18.97 | ↑ | 20.33 | ↑ | 9.69 | ↑ | 17.70 | ↑ | 19.05 | ↑ |
| PHOS | −2.97 | ↓ | 1.01 | ↑ | −2.22 | ↓ | 4.95 | ↑ | 9.26 | ↑ | 5.77 | ↑ |
| PLAT | 0.10 | ↑ | 6.68 | ↑ | 13.58 | ↑ | 19.05 | ↑* | 26.88 | ↑↑* | 35.08 | ↑↑* |
| PROT | 0.18 | ↑ | −5.24 | ↓ | −8.47 | ↓* | −4.27 | ↓ | −9.45 | ↓* | −12.53 | ↓* |
| PT | 1.00 | ↑ | 1.81 | ↑ | 4.56 | ↑ | 7.40 | ↑ | 8.26 | ↑ | 11.18 | ↑ |
| RBC | −3.14 | ↓ | −3.70 | ↓ | −0.53 | ↓ | −0.51 | ↓ | −1.08 | ↓ | 2.18 | ↑ |
| RETI | −4.63 | ↓ | 2.37 | ↑ | 16.77 | ↑ | −11.62 | ↓ | −5.14 | ↓ | 8.21 | ↑ |
| RETIRBC | −0.99 | ↓ | 7.10 | ↑ | 17.87 | ↑ | −10.60 | ↓ | −3.30 | ↓ | 6.43 | ↑ |
| SODIUM | 0.56 | ↑ | −0.07 | ↓ | 1.54 | ↑* | − 1.47 | ↓ | −2.09 | ↓* | −0.51 | ↓ |
| TRIG | 22.22 | ↑ | −8.08 | ↓ | 5.93 | ↑ | −27.60 | ↓↓ | −45.55 | ↓↓* | −37.25 | ↓↓* |
| WBC | −5.69 | ↓ | 1.07 | ↑ | 26.96 | ↑↑ | −7.93 | ↓ | −1.33 | ↓ | 23.94 | ↑ |

* Statistically significant compared to the control group (p < 0.05)

↑ Increase; ↓ Decrease; - No change. The number of arrows indicates the relative magnitude of percent change compared with that of the control group (1 arrow: <25%, 2 arrows: 25 ≤ 50%, 3 arrows: 50 ≤ 100%, 4 arrows: ≥100%).

Note: Clinical pathology parameters are presented using Standard for Exchange of Nonclinical Data (SEND) terminology to ensure data interoperability.

CCG, concurrent control group; VCG, virtual control group;

Abbreviations: ALB, albumin; ALBGLOB, albumin/globulin ratio; ALP, alkaline phosphatase; ALT, alanine aminotransferase; APTT, activated partial thromboplastin time; AST, aspartate aminotransferase; BASO, basophils (absolute); BASOLE, basophils/leukocytes (relative); BILI, total bilirubin; CA, calcium; CHOL, total cholesterol; CK, creatine kinase; CL, chloride; CREAT, creatinine; EOS, eosinophils (absolute); EOSLE, eosinophils/leukocytes (relative); GGT, gamma-glutamyl transferase; GLUC, glucose; HCT, hematocrit; HGB, hemoglobin; K, potassium; LGLUCLE, large unstained cells/leukocytes (relative); LGUNSCE, large unstained cells (absolute); LYM, lymphocytes (absolute); LYMLE, lymphocytes/leukocytes (relative); MCH, mean corpuscular hemoglobin; MCHC, mean corpuscular hemoglobin concentration; MCV, mean corpuscular volume; MONO, monocytes (absolute); MONOLE, monocytes/leukocytes (relative); NEUT, neutrophils (absolute); NEUTLE, neutrophils/leukocytes (relative); PHOS, inorganic phosphorus; PLAT, platelets; PROT, total protein; PT, prothrombin time; RBC, red blood cells; RETI, reticulocytes (absolute); RETIRBC, reticulocytes/erythrocytes (relative); SODIUM, sodium; TRIG, triglycerides; WBC, white blood cells.

**Supplementary Table S9. Comparison of percent changes in clinical pathology parameters of female rats in legacy study 2, CCG, and VCG.**

The table presents detailed directional changes, magnitude (percent change), and statistical significance of individual clinical pathology parameters in female rats of legacy study 2.

| Group | CCG vs. T1 | | CCG vs. T2 | | CCG vs. T3 | | VCG5 vs. T1 | | VCG5 vs. T2 | | VCG5 vs. T3 | |
| --- | --- | --- | --- | --- | --- | --- | --- | --- | --- | --- | --- | --- |
| Parameter | (%) | Change | (%) | Change | (%) | Change | (%) | Change | (%) | Change | (%) | Change |
| ALB | 2.99 | ↑ | −4.27 | ↓ | −5.11 | ↓ | −4.14 | ↓ | −10.90 | ↓* | −11.68 | ↓* |
| ALBGLOB | −0.99 | ↓ | 3.69 | ↑ | 3.42 | ↑ | −5.62 | ↓ | −1.16 | ↓ | −1.42 | ↓ |
| ALP | −10.44 | ↓ | −1.12 | ↓ | 27.09 | ↑↑ | −20.43 | ↓ | −12.15 | ↓ | 12.90 | ↑ |
| ALT | 86.49 | ↑↑↑ | −2.86 | ↓ | 24.21 | ↑ | 93.22 | ↑↑↑ | 0.64 | ↑ | 28.69 | ↑↑ |
| APTT | −7.14 | ↓ | −4.27 | ↓ | −8.42 | ↓ | 6.18 | ↑ | 9.46 | ↑ | 4.71 | ↑ |
| AST | 62.05 | ↑↑↑ | 7.38 | ↑ | 15.44 | ↑ | 33.12 | ↑↑ | −11.79 | ↓ | −5.16 | ↓ |
| BASO | 13.79 | ↑ | 44.83 | ↑↑ | 68.97 | ↑↑↑* | −4.35 | ↓ | 21.74 | ↑ | 42.03 | ↑↑* |
| BASOLE | 7.14 | ↑ | 0.00 | - | 0.00 | - | 33.33 | ↑↑ | 24.44 | ↑ | 24.44 | ↑ |
| BILI | 13.36 | ↑ | −7.96 | ↓ | −8.35 | ↓ | 19.59 | ↑ | −2.90 | ↓ | −3.32 | ↓ |
| CA | 2.97 | ↑ | −1.17 | ↓ | −1.07 | ↓ | 1.08 | ↑ | −2.99 | ↓ | −2.89 | ↓ |
| CHOL | −6.77 | ↓ | −15.46 | ↓ | −30.81 | ↓↓* | −4.18 | ↓ | −13.11 | ↓ | −28.89 | ↓↓* |
| CK | −12.80 | ↓ | 13.20 | ↑ | 13.80 | ↑ | −24.24 | ↓ | −1.65 | ↓ | −1.13 | ↓ |
| CL | 1.16 | ↑ | 0.19 | ↑ | 1.45 | ↑ | −0.76 | ↓ | −1.71 | ↓ | −0.47 | ↓ |
| CREAT | −7.21 | ↓ | −10.19 | ↓ | −5.96 | ↓ | 0.34 | ↑ | −2.88 | ↓ | 1.69 | ↑ |
| EOS | 45.10 | ↑↑ | 62.75 | ↑↑↑ | 49.02 | ↑↑ | −22.11 | ↓ | −12.63 | ↓ | −20.00 | ↓ |
| EOSLE | 38.30 | ↑↑ | 20.21 | ↑ | −5.32 | ↓ | 3.17 | ↑ | −10.32 | ↓ | −29.37 | ↓↓ |
| GGT | 259.37 | ↑↑↑↑ | 58.50 | ↑↑↑ | 55.62 | ↑↑↑ | 41.22 | ↑↑ | −37.71 | ↓↓ | −38.84 | ↓↓ |
| GLUC | 1.06 | ↑ | −6.70 | ↓ | 4.53 | ↑ | 8.09 | ↑ | −0.20 | ↓ | 11.81 | ↑ |
| HCT | 0.04 | ↑ | 0.50 | ↑ | 0.66 | ↑ | 5.38 | ↑ | 5.86 | ↑ | 6.03 | ↑ |
| HGB | 0.40 | ↑ | 0.53 | ↑ | −1.45 | ↓ | −2.78 | ↓ | −2.65 | ↓ | −4.56 | ↓ |
| K | 0.60 | ↑ | 7.90 | ↑ | 18.46 | ↑* | 7.74 | ↑ | 15.56 | ↑ | 26.87 | ↑↑* |
| LGLUCLE | 17.17 | ↑ | 32.32 | ↑↑ | 0.00 | - | 14.85 | ↑ | 29.70 | ↑↑ | −1.98 | ↓ |
| LGUNSCE | 25.93 | ↑↑ | 96.30 | ↑↑↑* | 59.26 | ↑↑↑ | −13.92 | ↓ | 34.18 | ↑↑ | 8.86 | ↑ |
| LYM | −3.21 | ↓ | 35.38 | ↑↑ | 46.98 | ↑↑* | −24.59 | ↓ | 5.47 | ↑ | 14.51 | ↑ |
| LYMLE | −4.30 | ↓ | −1.10 | ↓ | −3.00 | ↓ | −1.74 | ↓ | 1.55 | ↑ | −0.40 | ↓ |
| MCH | 0.67 | ↑ | 0.11 | ↑ | −2.12 | ↓ | −5.32 | ↓* | −5.84 | ↓* | −7.94 | ↓* |
| MCHC | 0.38 | ↑ | −0.06 | ↓ | −2.09 | ↓* | −7.80 | ↓* | −8.21 | ↓* | −10.07 | ↓* |
| MCV | 0.23 | ↑ | −0.04 | ↓ | −0.05 | ↓ | 2.74 | ↑ | 2.47 | ↑ | 2.45 | ↑ |
| MONO | 35.59 | ↑↑ | 40.11 | ↑↑ | 77.97 | ↑↑↑ | −2.04 | ↓ | 1.22 | ↑ | 28.57 | ↑↑ |
| MONOLE | 19.31 | ↑ | −0.31 | ↓ | 5.61 | ↑ | 16.06 | ↑ | −3.03 | ↓ | 2.73 | ↑ |
| NEUT | 31.98 | ↑↑ | 44.04 | ↑↑ | 93.90 | ↑↑↑* | −15.26 | ↓ | −7.51 | ↓ | 24.50 | ↑ |
| NEUTLE | 18.33 | ↑ | 3.08 | ↑ | 18.17 | ↑ | 3.78 | ↑ | −9.59 | ↓ | 3.64 | ↑ |
| PHOS | 2.49 | ↑ | 9.63 | ↑ | 16.89 | ↑* | 7.68 | ↑ | 15.19 | ↑ | 22.82 | ↑* |
| PLAT | 4.55 | ↑ | 4.38 | ↑ | 15.89 | ↑ | 34.43 | ↑↑* | 34.21 | ↑↑* | 49.01 | ↑↑* |
| PROT | 3.31 | ↑ | −5.32 | ↓ | −6.15 | ↓ | −2.13 | ↓ | −10.30 | ↓* | −11.10 | ↓* |
| PT | −0.72 | ↓ | 1.36 | ↑ | 4.87 | ↑ | −0.47 | ↓ | 1.61 | ↑ | 5.13 | ↑ |
| RBC | −0.22 | ↓ | 0.62 | ↑ | 0.75 | ↑ | 2.51 | ↑ | 3.37 | ↑ | 3.51 | ↑ |
| RETI | −5.79 | ↓ | −6.70 | ↓ | 32.94 | ↑↑ | −5.74 | ↓ | −6.64 | ↓ | 33.02 | ↑↑ |
| RETIRBC | −5.65 | ↓ | −7.08 | ↓ | 33.92 | ↑↑ | −7.97 | ↓ | −9.37 | ↓ | 30.61 | ↑↑ |
| SODIUM | 0.55 | ↑ | −1.24 | ↓* | −0.90 | ↓ | 0.07 | ↑ | −1.72 | ↓* | −1.37 | ↓* |
| TRIG | −6.67 | ↓ | −1.43 | ↓ | −23.21 | ↓ | −39.92 | ↓↓ | −36.55 | ↓↓ | −50.56 | ↓↓↓* |
| WBC | 2.98 | ↑ | 37.38 | ↑↑ | 53.83 | ↑↑↑* | −23.29 | ↓ | 2.33 | ↑ | 14.58 | ↑ |

* Statistically significant compared to the control group (p < 0.05)

↑ Increase; ↓ Decrease; - No change. The number of arrows indicates the relative magnitude of percent change compared with that of the control group (1 arrow: <25%, 2 arrows: 25 ≤ 50%, 3 arrows: 50 ≤ 100%, 4 arrows: ≥100%).

Note: Clinical pathology parameters are presented using Standard for Exchange of Nonclinical Data (SEND) terminology to ensure data interoperability.

CCG, concurrent control group; VCG, virtual control group;

Abbreviations: ALB, albumin; ALBGLOB, albumin/globulin ratio; ALP, alkaline phosphatase; ALT, alanine aminotransferase; APTT, activated partial thromboplastin time; AST, aspartate aminotransferase; BASO, basophils (absolute); BASOLE, basophils/leukocytes (relative); BILI, total bilirubin; CA, calcium; CHOL, total cholesterol; CK, creatine kinase; CL, chloride; CREAT, creatinine; EOS, eosinophils (absolute); EOSLE, eosinophils/leukocytes (relative); GGT, gamma-glutamyl transferase; GLUC, glucose; HCT, hematocrit; HGB, hemoglobin; K, potassium; LGLUCLE, large unstained cells/leukocytes (relative); LGUNSCE, large unstained cells (absolute); LYM, lymphocytes (absolute); LYMLE, lymphocytes/leukocytes (relative); MCH, mean corpuscular hemoglobin; MCHC, mean corpuscular hemoglobin concentration; MCV, mean corpuscular volume; MONO, monocytes (absolute); MONOLE, monocytes/leukocytes (relative); NEUT, neutrophils (absolute); NEUTLE, neutrophils/leukocytes (relative); PHOS, inorganic phosphorus; PLAT, platelets; PROT, total protein; PT, prothrombin time; RBC, red blood cells; RETI, reticulocytes (absolute); RETIRBC, reticulocytes/erythrocytes (relative); SODIUM, sodium; TRIG, triglycerides; WBC, white blood cells.

**Supplementary Table S10. Category-based reproducibility assessment of individual clinical pathology parameters.**

This table presents the assigned reproducibility category (1–5) for each clinical pathology parameter across legacy studies 1 and 2. The parameter-specific physiological range (<2% CV), derived from internal HCD was used as the threshold for determining category 4.

| **Parameter** | **Parameter-specific physiological range**  **(<2 CV%)** | | **Legacy study 1**  **(Male)** | **Legacy study 1**  **(Female)** | **Legacy study 2**  **(Male)** | **Legacy study 2**  **(Female)** |
| --- | --- | --- | --- | --- | --- | --- |
|  | **Male** | **Female** |  |  |  |  |
| **ALB** | ±9.7 | ±12.9 | Cat 4 | Cat 4 | Cat 4 | Cat 4 |
| **ALBGLOB** | ±13.0 | ±13.2 | Cat 1 | Cat 4 | Cat 4 | Cat 4 |
| **ALP** | ±49.0 | ±66.3 | Cat 4 | Cat 4 | Cat 1 | Cat 3 |
| **ALT** | ±82.9 | ±105.9 | Cat 2 | Cat 4 | Cat 1 | Cat 4 |
| **APTT** | ±20.9 | ±22.1 | Cat 4 | Cat 4 | Cat 4 | Cat 4 |
| **AST** | ±40.8 | ±64.5 | Cat 1 | Cat 1 | Cat 3 | Cat 4 |
| **BASO** | ±89.3 | ±85.3 | Cat 1 | Cat 4 | Cat 3 | Cat 4 |
| **BASOLE** | ±61.3 | ±74.0 | Cat 1 | Cat 4 | Cat 4 | Cat 4 |
| **BILI** | ±43.0 | ±40.9 | Cat 4 | Cat 1 | Cat 4 | Cat 1 |
| **CA** | ±9.3 | ±9.3 | Cat 4 | Cat 4 | Cat 4 | Cat 1 |
| **CHOL** | ±56.4 | ±53.7 | Cat 3 | Cat 2 | Cat 1 | Cat 1 |
| **CK** | ±68.5 | ±79.2 | Cat 1 | Cat 3 | Cat 1 | Cat 4 |
| **CL** | ±3.9 | ±4.5 | Cat 4 | Cat 4 | Cat 1 | Cat 4 |
| **CREAT** | ±20.5 | ±24.5 | Cat 4 | Cat 4 | Cat 1 | Cat 4 |
| **EOS** | ±84.7 | ±80.2 | Cat 4 | Cat 4 | Cat 4 | Cat 4 |
| **EOSLE** | ±70.2 | ±73.4 | Cat 3 | Cat 2 | Cat 3 | Cat 4 |
| **GGT** | ±129.3 | ±121.3 | Cat4 | Cat 4 | Cat 2 | Cat 4 |
| **GLUC** | ±51.9 | ±46.8 | Cat 4 | Cat 1 | Cat 1 | Cat 1 |
| **HCT** | ±11.5 | ±12.3 | Cat 4 | Cat 4 | Cat 4 | Cat 1 |
| **HGB** | ±8.5 | ±10.4 | Cat 4 | Cat 4 | Cat 2 | Cat 4 |
| **K** | ±36.0 | ±27.7 | Cat 4 | Cat 4 | Cat 4 | Cat 3 |
| **LGLUCLE** | ±79.1 | ±72.9 | Cat 4 | Cat 2 | Cat 3 | Cat 4 |
| **LGUNSCE** | ±97.1 | ±104.0 | Cat 4 | Cat 3 | Cat 3 | Cat 4 |
| **LYM** | ±56.5 | ±67.7 | Cat 4 | Cat 3 | Cat 1 | Cat 3 |
| **LYMLE** | ±13.4 | ±18.4 | Cat 2 | Cat 1 | Cat 1 | Cat 4 |
| **MCH** | ±8.5 | ±7.7 | Cat 1 | Cat 2 | Cat 4 | Cat 4 |
| **MCHC** | ±7.8 | ±8.0 | Cat 4 | Cat 4 | Cat 2 | Cat 4 |
| **MCV** | ±8.5 | ±8.2 | Cat 2 | Cat 4 | Cat 1 | Cat 4 |
| **MONO** | ±86.7 | ±95.6 | Cat 2 | Cat 4 | Cat 4 | Cat 4 |
| **MONOLE** | ±64.4 | ±80.0 | Cat 1 | Cat 1 | Cat 4 | Cat 1 |
| **NEUT** | ±76.3 | ±102.2 | Cat 4 | Cat 4 | Cat 2 | Cat 4 |
| **NEUTLE** | ±64.3 | ±99.2 | Cat 3 | Cat 1 | Cat 1 | Cat 4 |
| **PHOS** | ±21.7 | ±29.0 | Cat 4 | Cat 4 | Cat 4 | Cat 1 |
| **PLAT** | ±29.8 | ±30.7 | Cat 4 | Cat 4 | Cat 3 | Cat 3 |
| **PROT** | ±11.1 | ±13.2 | Cat 4 | Cat 2 | Cat 4 | Cat 4 |
| **PT** | ±14.6 | ±10.3 | Cat 1 | Cat 4 | Cat 1 | Cat 1 |
| **RBC** | ±11.2 | ±12.1 | Cat 4 | Cat 4 | Cat 4 | Cat 4 |
| **RETI** | ±34.9 | ±49.7 | Cat 4 | Cat 4 | Cat 4 | Cat 1 |
| **RETIRBC** | ±37.1 | ±51.9 | Cat 4 | Cat 4 | Cat 4 | Cat 1 |
| **SODIUM** | ±3.4 | ±2.9 | Cat 4 | Cat 4 | Cat 4 | Cat 2 |
| **TRIG** | ±139.9 | ±11.2 | Cat 4 | Cat 4 | Cat 4 | Cat 3 |
| **WBC** | ±52.5 | ±163.2 | Cat 4 | Cat 3 | Cat 4 | Cat 4 |

HCD, historical control data; CV, coefficient of variation;

Abbreviations: ALB, albumin; ALBGLOB, albumin/globulin ratio; ALP, alkaline phosphatase; ALT, alanine aminotransferase; APTT, activated partial thromboplastin time; AST, aspartate aminotransferase; BASO, basophils (absolute); BASOLE, basophils/leukocytes (relative); BILI, total bilirubin; CA, calcium; CHOL, total cholesterol; CK, creatine kinase; CL, chloride; CREAT, creatinine; EOS, eosinophils (absolute); EOSLE, eosinophils/leukocytes (relative); GGT, gamma-glutamyl transferase; GLUC, glucose; HCT, hematocrit; HGB, hemoglobin; K, potassium; LGLUCLE, large unstained cells/leukocytes (relative); LGUNSCE, large unstained cells (absolute); LYM, lymphocytes (absolute); LYMLE, lymphocytes/leukocytes (relative); MCH, mean corpuscular hemoglobin; MCHC, mean corpuscular hemoglobin concentration; MCV, mean corpuscular volume; MONO, monocytes (absolute); MONOLE, monocytes/leukocytes (relative); NEUT, neutrophils (absolute); NEUTLE, neutrophils/leukocytes (relative); PHOS, inorganic phosphorus; PLAT, platelets; PROT, total protein; PT, prothrombin time; RBC, red blood cells; RETI, reticulocytes (absolute); RETIRBC, reticulocytes/erythrocytes (relative); SODIUM, sodium; TRIG, triglycerides; WBC, white blood cells.
